# World Cafés as a participatory approach to understanding research agendas in primary care with underserved communities: reflections, challenges and lessons learned

**DOI:** 10.1186/s40900-023-00509-3

**Published:** 2023-10-28

**Authors:** Carmel McGrath, Mari-Rose Kennedy, Andy Gibson, Samira Musse, Zahra Kosar, Shoba Dawson

**Affiliations:** 1 NIHR Applied Research Collaboration West, University Hospitals Bristol and Weston NHS Foundation Trust, Bristol, UK; 2 NIHR Health Protection Research Unit in Behavioural Science and Evaluation, Population Health Sciences, Bristol Medical School, University of Bristol, Bristol, UK; 3 Faculty of Health and Applied Sciences, School of Health and Social Wellbeing, University of West England, Bristol, UK; 4 People in Health West of England, University of the West of England, Bristol, UK; 5 Barton Hill Activity Club, Bristol, UK; 6 Bristol Medical School, University of Bristol, Bristol, UK; 7 Somali Resource Centre, Bristol, UK; 8 Centre for Academic Primary Care, Bristol Medical School, University of Bristol, Bristol, UK

**Keywords:** World Cafés, Participatory approaches, Primary care, Underserved communities, Public involvement, Research agendas

## Abstract

**Background:**

Certain communities are underserved by research, resulting in lower inclusion rates, under researched health issues and a lack of attention to how different communities respond to health interventions. Minoritised ethnic groups are often underserved by research and services. They experience health inequalities and face significant barriers to accessing health services. It is recognised that new approaches are needed to reach underserved communities and make research more relevant. The purpose of this work was to utilise World Cafés, a participatory method, to explore research agendas with diverse communities.

**Methods:**

Two World Cafés were conducted as research agenda setting activities with individuals from minoritised ethnic communities in Bristol, UK. World Café 1 explored Black and Asian women’s perspectives about supporting mental health. World Café 2 with men from the Somali community, focused on prostate cancer. Community members co-developed the focus and questions of each World Café and were also instrumental in recruiting individuals to the sessions and facilitating discussions, including translation. Audio and written records were made of the discussions and from these key issues about each topic were identified, and a visual representation of the discussion was also generated. These were shared with participants to check for accuracy.

**Results:**

Community members identified a range of issues that are important to them in relation to mental health and prostate cancer, including barriers to help seeking and accessing primary and secondary care, ideas for service improvements and a need for health information that is accessible and culturally relevant.

**Conclusions:**

World Cafés are a flexible method that can be successfully adapted for research agenda setting with individuals from minoritised ethnic communities. The role of community members in co-developing the focus of sessions, recruiting community members and co-facilitating sessions is crucial to this success. The discussions at both World Cafés provided a rich insight into the experiences of participants in relation to the topics mental health and prostate cancer and identified issues that are important to these communities that will be followed-up with communities, researchers and clinicians to co-develop research and service improvement strategies.

**Supplementary Information:**

The online version contains supplementary material available at 10.1186/s40900-023-00509-3.

## Abstract (plain language summary)

People from minoritised ethnic groups can have difficulty getting health care. They may also be left out of health research. There is a need to include a more diverse group of people in health research. World Cafés are an accessible way to listen to people by getting them to talk to each other about different questions and topics.

We did this work to see if World Cafés are a helpful way to talk to people from minoritised ethnic groups about health. We wanted to learn what things are important to people around mental health and prostate cancer. We also wanted to see if designing and running the World Café with people from the same communities as people taking part in the World Cafés is a good way to do this. We worked together with people from racial and ethnic minorities to:

- Choose questions and topics,
- Ask people to take part
- Get people talking and write down what was said

We learned that planning and running World Cafés with people from the same communities as the people taking part was very helpful. The people taking part in the World Cafés said that it can be difficult to get help and information about their health. They gave lots of reasons for this. They also had ideas to improve healthcare.

Now that we have this information, we will work with the people who took part in and helped run the World Cafés, along with, doctors and researchers to take their ideas for improvements forwards.

**Supplementary Information:**

The online version contains supplementary material available at 10.1186/s40900-023-00509-3.

## Introduction

The term underserved communities reflects the reality that research needs to provide a better service to people in certain communities. Who these people are will differ depending on the research question being asked, the relevant population and the type of intervention under consideration [1]. Underserved communities are frequently drawn from people on lower incomes, in minoritised ethnic groups or people who suffer discrimination and prejudice due to other characteristics e.g., sexual identity and orientation. An underserved community can be defined as any community where there is a lower inclusion rate in research compared to population estimates, experience of a healthcare burden unmatched by the volume of research, or where there are important differences in how a group responds to or engages with healthcare interventions compared to other groups. For a more detailed discussion see Improving inclusion of under-served groups in clinical research: Guidance from INCLUDE project NIHR (2022) [1].

It is known that underserved communities, including those from minoritised ethnic groups, experience health inequalities in terms of both morbidity and mortality and face significant barriers to accessing health services [2]. This has been further highlighted by the COVID-19 pandemic. In comparison to the general population, people from certain minoritised ethnic groups and people on low incomes experienced poorer health outcomes and inadequate quality health care [3]. Additionally, physical and other barriers including language, literacy, patient perceptions, staff attitudes, poorer patient-professional communication and mistrust of services [4] can lead to exclusion from health and social services [4–7]. Participation, involvement, and engagement in research do not always reflect the diversity of the general population. This means that research findings have limited relevance and generalisability and is therefore both a scientific and ethical problem [8, 9]. More recently, the NIHR INCLUDE project developed guidance to improve research for underserved groups; it emphasises the need for strong community engagement, co-development, and working with communities to set research agendas and develop interventions [1]. To maximise the benefits of research, it is important to work in partnership with underserved communities to develop an understanding of what research agendas matter to them. There is a need for shift in practice from researcher-led approaches to co-designed approaches. There are a variety of methods that can be used to develop agendas in health research with patients and clinicians [10]. However, some of these approaches are time consuming and may not be accessible to underserved communities. McFarlane et al. suggest that participatory methods, specifically World Cafés, offer a valuable method in which to explore research agendas with under-served communities [10].

World Cafés are a flexible participatory method that have been used widely to explore issues that are important to communities [11] and are an inclusive tool to engage with diverse communities [12]. Central to the approach is fostering an environment where communities involved can have “conversations that matter”, through creating an environment where people can communicate constructively, enabling access to “collective intelligence and creating innovative possibilities for action” (p. 3) [11]. Participants in World Cafe discussions are considered “experts in their own lived experience and experiential knowledge” (p. 279) [10]. The aim is to encourage a wide range of perspectives, rather than necessarily reaching consensus [10]. In this paper, we report on two World Cafés that were used to identify research agendas that are important to diverse communities and co-develop research ideas for future primary care research. The World Cafés focused on two main areas; mental health and prostate cancer. In the wider literature, it has been reported that minoritised ethnic groups in the UK have faced persistent disparities in mental health and access to care, which have been documented for over fifty years. It has been suggested that equitable care depends on the active involvement of people with the lived experience from minoritised ethnic groups in the design and development of mental healthcare and services.

In relation to prostate cancer, evidence has shown that Black men are 2–3 times more likely to be diagnosed with prostate cancer compared to White men. Despite this increased risk it has been reported that Black men are more likely to experience delayed referrals and receive less intensive forms of treatment [13]. These differences in care and treatment have been recognised and there are active efforts being made to address the lack of evidence and research into racial disparities in this area. We therefore identify key issues raised as important by these communities and reflect on the learnings to make key recommendations for those who wish to use similar participatory approaches in the future.

## Methods

### Context and design

World Cafés (WCs) enable communities to get together in an informal setting to discuss an issue in small table groups. World Cafés support open conversation and enable people to talk about things that matter to them [10]. The method facilitates community change by hearing and exchanging ideas of as many community members as possible on topic areas that matter to them. The traditional World Café approach is outlined in Table 1. A benefit of World Café is that it can be run in several languages concurrently, making it ideally suited to working with minoritised ethnic communities where English may not be the first language of participants. A particular strength of World Cafés is cross-pollination of ideas through rounds of discussions, facilitating information exchange in an equitable and nonthreatening manner [14].

**Table 1:** The traditional process used for conducting World Cafés

Process—The World Café Foundation (http://www.theworldcafe.com/) provides a toolkit that includes an integrated set of design principles and graphics that are freely available. The basic model incorporates the following elements:
1. Setting—an informal, café style environment (e.g., a community centre)
2. Welcome and introduction—the host puts participants at ease through a warm welcome and refreshments, overview and ground rules for participation
3. Small-Group Rounds—20-min rounds of conversation between 5 and 6 participants
4. Questions—a key question is discussed in each round, which may build upon one another in subsequent rounds. We anticipate asking 4–5 questions which will be developed in collaboration with our expert panel
5. Harvest—each group is invited to share the results of their conversations with the wider group, which are reflected visually (e.g., through graphic recording in front of the room by an artist)

We conducted two WCs in the city of Bristol, UK. WC1, focusing on supporting mental health, took place in July 2022 and was predominantly attended by women from local African-Caribbean communities. WC2, focusing on prostate cancer, took place in September 2022 and was attended by men from the local Somali community. Both WCs were held at the University of Bristol Micro-campus, in Barton Hill, and were facilitated and translated by community facilitators. Barton Hill is an inner-city area with a diverse population and ranked 28th most deprived in the Bristol City Council 2019 Measure of Indices of Deprivation [15].

### WC1: supporting mental health and wellbeing—women’s group

Prior to WC1, SD and CM met ZK and SM. ZM and SM are community leaders and they were involved in recruiting community members and co-facilitating the two WCs. ZM and SM introduced SD and CM to eight women from the community who registered an interest in supporting the World Cafés, to determine the topic area that were a priority to these communities and relevant questions to ask. From this discussion, three questions were developed focusing on mental health and wellbeing (see Table 2 below). The areas explored included: how we can best support people to seek medical advice, including the role of families in caring for and supporting an individual to access services.

**Table 2:** Questions for World Café 1: mental health

1	How do we encourage and support people to seek medical help/advice as soon as possible?
2	What role can family members and friends play in care for the individual with mental health problems, and how can they be supported?
3	What would you like from general practice/GPs in terms of support in accessing services?

We also recruited facilitators from this planning session. We provided an overview of World Café method using the steps outlined in Table 1 to explain the approach and offered our support to facilitate the discussions on the day. For example, it was agreed that it would be helpful if CM and SD were present during the discussions to help with writing the post it notes while they concentrated on asking the questions and ensuring the participants could all have opportunities to speak.

As part of this pre-meeting, we also talked through strategies for supporting the people involved in the World Cafés given the sensitive topic area. For each of the World Cafés, ground rules were set out to ensure respectful communication and that participants understood what support was available during the World Cafés. A signposting resource was developed and shared with the attendees’ post WCs. Women were recruited via different networks from Asian, Black African, and Caribbean communities locally.

### WC2: prostate cancer and experiences of accessing primary care for this—men’s group

Prior to WC2, SD discussed the area of focus and developed questions with ZK and SM. The chosen topic explored understanding Somali men’s views on accessing GP services for prostate cancer testing, barriers to accessing GP services and experiences of using community pharmacies (see Table 3. SD, CM and MK took notes in this session, and a male community facilitator who participated, and helped interpret the session. Men were recruited via ZK and SM’s community networks.

**Table 3:** Questions for World Café 2: prostate cancer

1	What are your views about seeing a GP to discuss examination and testing for prostate cancer?
2	What will help or hinder you from using this service? How does your family influence this?
3	What is your experience of community pharmacies? Have you used them?

### Running the Cafés

On the day of each WC, individuals were welcomed and invited to join groups of 6–8 individuals at separate tables with two facilitators on each table (one to take notes, one to facilitate the conversations). For both Cafés, we had non-English speakers and experienced community facilitators helped translate and back-translate the discussions to enable note taking. Before the discussions commenced, the facilitators explained the WC process to the group by introducing the goals of the session and setting ground rules.

One question was posed for each round of discussion, followed by a summary of discussions before proceeding with subsequent rounds of questions. Community facilitators helped to keep the groups at the table focused on the topic and encouraged participation from everyone. Individuals could contribute verbally or by writing their thoughts on post-it notes. Community facilitators helped translate questions and discussion points to non-English speaking individuals. In WC1, multilingual scribes sat at tables with corresponding multi-lingual individuals to summarise their discussions in English and facilitate discussions with the rest of the group. In WC2, discussions were solely in Somali. Individuals moved tables between questions to interact with different people. Where individuals spoke in different languages to English, they stayed in the group with a facilitator and scribe who could speak the same language.

In addition to written notes, discussion groups in WC1 were audio recorded with everyone’s permission. Given the sensitive nature of the topic area and to facilitate open conversation, discussions in WC2 were not recorded. This was decided in advance through consultation with the community facilitators, who explained that Somali men did not tend to discuss prostate related issues amongst themselves or with family members as it was something they considered private. Due to this reason, we also did not rotate tables at the end of each round to enable open discussion within the group. For the two WCs, we did not rank priorities at the end as one typically would using such a methodology. This is because the way the questions were designed meant we took a more exploratory approach, as we were not focusing on priorities in the questioning. At the end of the WCs, contributors were informed about follow-up opportunities to be involved in shaping research in this area, based on the discussions and topics raised.

### Post Cafés

Following the Cafés, written and audio records of the discussions were reviewed by CM, MK and SD, who ensured that these were anonymised and developed a list of key issues. A summary of the key points raised in each Café was provided to an artist who produced a visual storyboard of discussions to facilitate accessibility of the summarised discussions and to promote further conversation. The full report of the discussions including key points and artistic illustrations from the WCs were shared with all contributors by ZK and SM via WhatsApp for sense checking and accuracy. Comments from community facilitators and contributors were used to check and further clarify or contextualise these summaries where necessary. ZK and SM also had further opportunity to provide input into the identified key issues during the development of this manuscript. We completed the GRIPP2 checklist [16] for public involvement activities which can be found in Additional file 1: Appendix 1.

## Results

### WC1: mental health

Twenty-four (n = 24) women: n = 22 Black African and Caribbean, n = 1 South Asian, n = 1 East Asian/other Asian attended WC1..

After examining the discussions to the first question: *‘How do we encourage and support people to seek medical help/advice as soon as possible?’*, we identified two key issues: (1) Creating specialist roles to act as a bridge between the community and healthcare services and (2) Changes to healthcare provision.

#### Issue 1: creating specialist roles to act as a bridge between the community and healthcare services

The discussions identified a need for role models, who are people from the communities specifically providing support for the mental health needs of the relevant communities. The groups identified various reasons why this role was considered important and thought it would encourage communities to seek help and advice. They recognised that cultural barriers were not being addressed as well as existing stigma associated with mental health more generally. As a result, these issues prevented people from seeking support. To overcome them, the women discussed the important characteristics required of the person providing support. The groups felt that it needed to be someone who could be trusted by the community and who would be non-judgemental, empathetic and sensitive. They also recognised the various roles that a health specialist may need to undertake including advocacy, signposting and education. The women felt that an advocacy role included supporting the individuals to take their medications, involvement in decisions about their mental health and supporting them to take the first step in accessing services.

#### Issue 2: changes to healthcare provision

Issues relating to knowledge, information and delivery of healthcare services that were considered barriers and preventing communities from accessing support were identified. For example, there was a clear need to improve signposting to relevant services because currently there was a lack of knowledge about the range of services already in existence. The groups felt that individuals found it challenging to navigate the complex system and understand how they could access various services. It was believed that training, education and processes were required within organisations to simplify the journey of accessing treatment and support. Additionally, it was considered important to provide education around mental health to the individuals and this included avoiding jargon and complex terminology.

The women acknowledged that there were issues with how the current primary care systems operate, which created barriers in access to mental health support for communities. For example, there were concerns that support from care services had worsened since the COVID-19 pandemic. While they recognised a lack of services, individuals felt that GPs had a responsibility to ensure individuals were signposted to the relevant professionals in a timely manner.

Ways to overcome these barriers included the investment of additional funding from the government for services to support mental health and creating ‘GP events’ in the community e.g., drop-ins or socials. It was envisioned that at these events, it would be possible to have discussions around mental health, whilst maintaining the ability to socialise and connect with others. Individuals felt that the healthcare system could be more proactive in prevention by implementing mental health reviews and checks. They also suggested liaising with wider services to ensure that the individuals’ care is holistic, and person centred. Some strategies that could be undertaken by community members to support individuals with accessing mental health support included being aware of other people’s needs, encouraging people to self-care, sharing information. Additionally, community members could assist by signposting to services and listening to individuals without being judgmental.

Response to the second question: *‘What role can family members and friends play in care for the individual with MH problems and how can they be supported?’* resulted in two further issues: (3) Taking a proactive, holistic and supportive approach and (4) Education, information and communication and community support.

#### Issue 3: taking a proactive and supportive approach

The discussions were centred on family members and friends working together with the individual to support them with their mental health. In some cases, this involved the family members or friends creating time and spaces for conversations around mental health as well as helping to proactively signpost and create strategies for help and support*.* The groups also mentioned the importance of the family member or friend providing support to the individual being the ‘right’ person as family/friends may not always be the best placed to provide support. They also highlighted some important characteristics required of the individual providing support, i.e., there was consensus that the person seeking support must fully trust the person providing support and expected them to be empathetic, sensitive and non-judgemental as people may feel judged due to the stigma associated with mental health. In other discussions, it was recognised how these important characteristics and behaviours of the individual providing support could help the person seeking support to feel at ease when talking about mental health.

When discussing strategies for seeking mental health support, the women emphasised the significant role of religion and belief systems. Some religions use traditional healing methods and spirituality, and individuals may use such support from religious leaders before seeking help from medical professionals.

#### Issue 4: education, information and communication and community support

Individuals also recognised the support that could be beneficial to family and friends. It was considered that they (family and friends) may not have sufficient information to feel confident in supporting an individual with their mental health needs. Specific information on the behaviours of people requiring support for mental health was considered highly valuable. The importance of communication between family, healthcare providers and the individual requiring support was highlighted. The groups mentioned that support could be provided by the individual’s wider community. In some conversations, the women alluded to specific examples of where additional education and information on mental health and mental health first aid training would be important for the community and in places of worship:

The third question: *‘What would you like from general practice/GPs in terms of support in accessing services?’* resulted in two final issues: 5) Individualised care and 6) Improved support services.

#### Issue 5: individualised care

Recommendations that primary healthcare services including GPs need to provide bespoke care to individuals who are requiring support for their mental health were discussed. The suggestions included extending GP appointment times based on individual needs, providing language accommodations (e.g., employing a translator), and supporting the individual in advocating for their care and treatment decisions and the need for early intervention.

The need for healthcare professionals and staff (including GP receptionists) to be provided with cultural sensitivity training was highlighted with participants highlighting a preference for speaking to ‘someone like them’ to ensure that cultural context was accounted for. Individuals raised the importance and responsibility of healthcare professionals to prioritise conversations about mental health.

#### Issue 6: improved support services

Individuals discussed ways to improve services more generally. For example, providing increased funding, support and access to mental health services and additional training and support to communities so they can better understand how to access support. The women considered it important to raise further awareness of the existing support available: better signposting to community support services, providing accessible information leaflets, better advertising about what services GPs can offer and other services. Additional areas of improvement included, reducing referral times and extending GP appointments, particularly when discussing a person’s mental health and wellbeing as these topics require additional time to fully understand people’s needs.

**Fig. 1:**
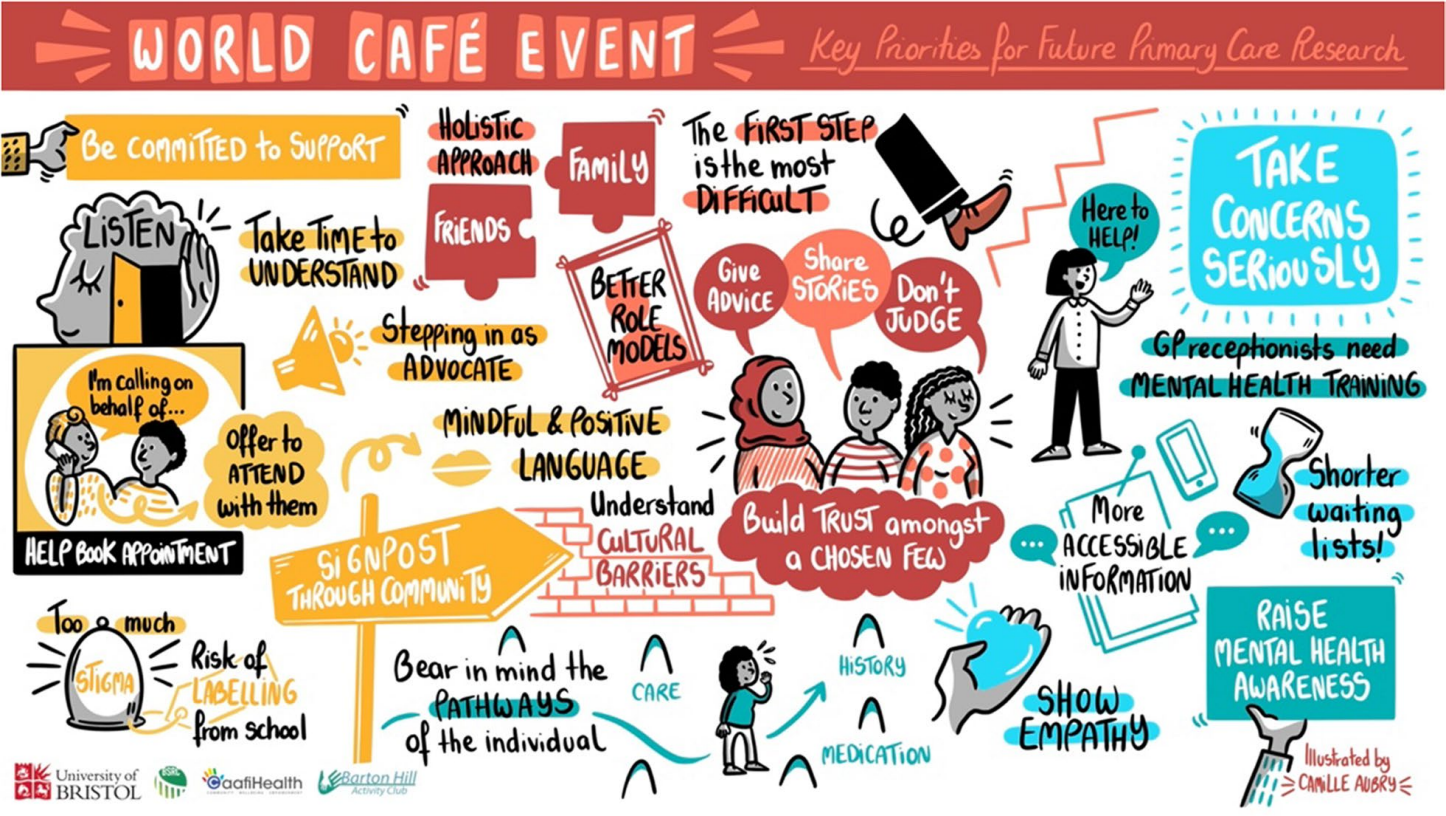
A visual illustration of notes from World Café 1

The women also suggested improvements to provide equitable, inclusive and culturally sensitive care. For example, the staff who deliver healthcare services, including receptionists, could benefit from training including cultural competency training. Some felt that there should be more services based in GPs that could provide translation including translating the initial GP sign-up forms. Additionally, providing patients with a choice of their GP, e.g., male or female GPs as this would enable patients to feel at ease during their appointments and get more out of their appointments. Lastly, integrating specialist teams, clinics and drop-in sessions into communities would help to support people with their mental health as this would make it safer and easy for people to engage and access care (Fig. 1).

### WC2: Somali men’s views on prostate cancer

Twenty-two (n = 22) Somali men attended WC2.

In response to the first question: *What are your views about seeing a GP to discuss examination and testing for prostate cancer?* Three key issues were identified: (1) Confusion about diagnosis and treatment; (2) Positive experiences about visiting GPs and (3) Barriers to visiting GPs.

#### Issue 1: confusion about diagnosis and treatment

Several men described visiting the GP for urinary and prostate related problems, however it was evident that some were confused about their diagnoses and whether their problem related to prostate cancer or not. This was due to a lack of effective communication or information provision from GPs to the men about symptoms, testing and treatments related to their care. Individuals also drew on their experiences of visiting the GP about other health problems such as diabetes. To meet their informational needs some used leaflets, the internet and YouTube videos created by healthcare professionals who spoke the same language as them. In turn this made it more likely that they would follow that advice. On the other hand, some men seemed knowledgeable about their healthcare, diagnoses and treatments and had some awareness about prostate cancer.

#### Issue 2: positive experiences and views about visiting GPs and specialists

Individuals reported feeling positive about visiting their GP including a sense of respect and trust in them because of health-related expertise and knowledge. Some men also recognised the value of visiting GPs because of their role in helping access secondary healthcare services. Several men described feeling comfortable in visiting their GP to talk about their health in general. However, others seemed to speak more specifically about experiences related to prostate cancer testing and screening services, where they thought that it was good to see a doctor and get tested.

The groups also discussed the roles of pharmacists who they saw as distinct from doctors, and not necessarily professionals who they would approach for consultation about serious health concerns. Pharmacists’ expertise was understood as dispensing medicine and dealing with minor ailments, so that consultation about serious health conditions was not viewed as being part of their remit.

#### Issue 3: barriers to visiting GPs

Barriers to visiting GPs were also discussed, with men reporting not visiting their GP despite experiencing health problems, highlighting a need to explore the potential reasons behind this behaviour to try and improve help-seeking. In relation to prostate cancer related problems, it was evident that at least some men experienced shame and embarrassment due to the nature of the disease and examination procedure which could pose a barrier. Another barrier to help seeking was that they felt that their GPs lacked action or failed to provide sufficient information, or they had experienced delays after visiting their GP. Individuals also reported going abroad to seek help for health problems including for prostate cancer and suspected diabetes.

In response to the second question: *What will help or hinder you from using this service? How does your family influence this?*, the men mostly discussed further barriers and facilitators experienced by individuals in relation to vising their GP or the pharmacist, rather than the influence of family and friends; so this aspect could be explored further in the future. Issues identified were: (3) Barriers to visiting GPs (continued); (4) Men’s reasons for not seeking help; (5) Talking to friends and family.

#### Issue 3: barriers to visiting GPs (continued)

Whilst the focus was about family influences that may help or hinder Somali men from using GP services, the conversation tended to gravitate towards barriers that were seemingly unrelated to these influences. Building on their previous discussions about barriers men described how they found accessing the GP difficult due to long waiting times and bureaucracy in making appointments. There were also similar concerns voiced about visiting pharmacists. Additionally*,* the process of completing online appointment forms was discussed as a barrier to accessing GPs. The forms were considered lengthy and repetitive, and there were concerns that there may be additional challenges in completing the forms for those who may not speak English and for older adults who may be less familiar with using digital technology. This was considered to prevent people from being able to make a choice about their care and ability to access services.

Individuals also expressed concerns about poor communication between healthcare professionals—hospital consultants, GPs and pharmacists and worried that it could result in mistakes and/or delays in receiving results and treatment. This lack of communication between service providers led some men to resort to alternative treatments such as using traditional medicine or seek help from abroad.

#### Issue 4: men’s reasons for not seeking help

The groups discussed some reasons why they might not seek help about prostate cancer from their GP as some had not heard about the disease before and others suggested a need for more information and awareness raising. Individuals explained that men from their community might not see the GP because of a lack of awareness about the disease and symptoms. Also, if men were not experiencing pain, they might not realise that they could have a health problem. This explicit desire for further information and advice from healthcare providers about prostate cancer, symptoms, stages of the disease and treatment by the men suggests that a review of current information provision in consultation with Somali men might be helpful to ensure that information is accessible and culturally relevant to this community. Some men also identified that whilst they might approach their GP for prostate problems, they might not be willing to undergo some examinations (for example, a rectal examination), due to the sensitive nature of these. They thought that this reticence could hinder some men’s help seeking.

Some men reported their experiences of visiting a doctor in relation to diabetes where they felt that they had been given false-positive results, causing unnecessary fear and worry. They attributed the mistakes in their diagnoses to a lack of communication on behalf of their doctor to check with them their sugar consumption prior to testing. Therefore, some feared that visiting their GP might cause unnecessary worry because of the unknown and worried about health problems that might be identified, and the consequences of this on them and their family.

#### Issue 5: talking to friends and family

In discussion of this question, there were mixed views about talking to family and friends about health and little discussion about their influence regarding helping or hindering service use. Some men said that they might speak about health concerns with close friends, and it was apparent that individuals did talk to others about their health concerns. Several reasons for not talking to friends and family about prostate cancer and health more generally as people lacked awareness about prostate cancer and consequently cannot talk about it easily and in other cases, they described their health as a private issue.

The discussions in response to the third question: *“What is your experience with community pharmacies? Have you used them?”* revealed that there seemed to be limited awareness about, and experience of community pharmacists amongst the men. Two issues were identified (6) the role of pharmacists, and (7) problems using pharmacists.

#### Issue 6: the role of pharmacists

A prevailing view amongst the men was that a pharmacist’s role was to primarily supply medication, however, there was also some understanding of other services that pharmacists could provide such as blood pressure checks or vaccinations. Certainly, the men seemed unaware of the role of community pharmacists, so raising awareness of these could be a potential route to improve access and reduce delays or provide outreach services (where relevant) in the Somali community.

#### Issue 7: problems using pharmacists

The men outlined several problems that they had experienced in using pharmacists. This included delays in receiving medication due to stocking issues and poor communication from pharmacists with regards to this made them unsure about when they would eventually receive their medication. They also felt that problems with communication between GPs and hospitals that could cause confusion in relation dispensing their medication.

**Fig. 2:**
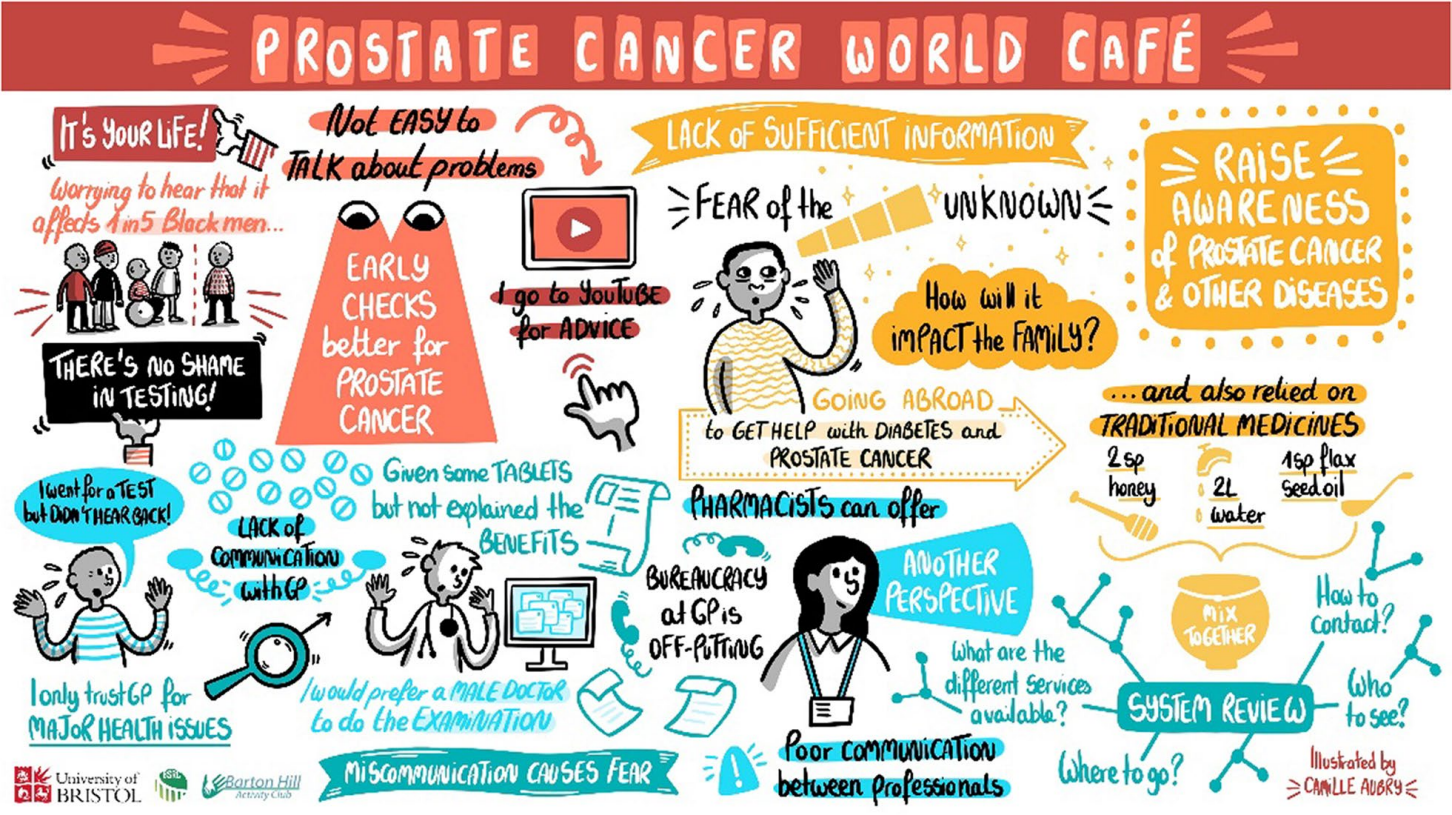
A visual illustration of notes from World Café 2

Finally, there were discussions around how cheaper brands of medication meant poorer quality and less effective. Individuals gave examples where their doctor and/or pharmacist had swapped the brand of their medication, and they were not satisfied with the change despite it being the same drug. This view seemed prevalent, but not all were concerned because they felt that there was choice available to them in terms of medication and pharmacists (Fig. 2).

## Discussion

Our paper reports the discussions that took place over two World Cafés , aiming to explore key research agendas in primary care focused in two main areas: mental health and prostate cancer.

Across the two World Cafés there were some overlapping issues identified. Both groups recognised the need for healthcare services to improve and adapt how they signpost and communicate information to communities about the range of existing services in addition to GPs. The women and men also highlighted the importance of offering people longer appointment times as well as providing people with a choice about who provided their care and treatment. From the discussions, it was evident that the groups placed value on the continuity of care and receiving care from someone they could trust and feel understood by.

Some of the issues raised in the discussions would benefit from further research whilst others were more related to service improvement, e.g., the provision of interpreters. There is no reason why World Café participants would make a distinction between issues which might benefit from further research and some which are suggestions for service improvement. Locally we are developing links with our Integrated Care System which also carries out engagement and research activities. Developing relationships of this kind will help us to take forward issues in the most effective way possible. Working across organisational boundaries is important in developing an effective response to the issues raised by underserved communities who have repeatedly expressed the problems of consultation fatigue that is generated by organisations working in silos.

Below we focus on how some of the issues raised in the WC can be taken forward as research projects.

### Recommendations for future research

The issues raised by the groups are important and timely; one example of further research that could be developed in light of the community’s discussions relates to the NHS long-term plan, which has recently detailed plans to address the existing divide between primary and community health services [17] by introducing social prescribing link workers into primary care networks (PCNs) [18].

The model and terms used to describe social prescribing link workers (also referred to as community link workers, community connector or navigators) may vary across care settings and organisations. According to the NHS, they are based in GP practices and their main purpose is to ensure people are connected to relevant activities, groups, and services in their communities in order to ensure their practical, social and emotional needs affecting their health and wellbeing are met [18, 19]. A recent development of this initiative has been the requirement for PCNs to work with a population experiencing health inequalities to proactively offer social prescribing interventions.

Revisiting the key issues raised by the World Cafés relating to the importance of providing individualised care and better signposting to additional services, it is apparent that the link worker role could be a solution. However, despite the social prescribing link workers initiative existing in the areas that the World Cafés took place, it is evident that this resource is currently unknown and underutilised, despite NHS targets to have at least 900,000 people referred to social prescribing by 2023/24 [18].

The current evaluations of social link prescribers have been inconsistent and undertaken using heterogeneous designs and methods and therefore have resulted in inconclusive evidence of their success in practice [19]. We suggest that the evaluation of social prescribing link workers should be explored through further research to better understand how they can be visible, responsive and engaged with their local communities. This should include well-conducted studies designed to measure the impact of these initiatives.

Moreover, the World Café participants identified the need for translators during GP appointments, translated information materials and longer GP appointments. The topic of GP appointment times has been discussed more widely. Currently, the average length of GP consultations in the UK is 9.2 mins, however, it has been recognised that this is not sufficient to allow GPs to deliver holistic care to patients and discuss their physical, psychological, and social needs [20]. Furthermore, during the World Cafés, it was highlighted that where translation is required for a patient, this would naturally add additional time to allow the patient to fully explain their health needs However this additional time required may not always be accounted for in practice. Despite the recognition of this important issue, there are seldom studies exploring the benefits of offering and sharing decisions over appointment lengths with patients [21]. We would therefore recommend that future research is conducted in this area to improve the effectiveness and efficiency of GP appointments.

We also suggest that the existing roles of social link prescribers are revisited before expanding this initiative further to explore if there is scope to develop their roles and responsibilities. These could include being present at community events, advocating for longer GP appointment times and ensuring translators are present and translated information materials are available when required. It is unclear if these are currently roles of social prescribing link workers, however the inclusion of these roles may lead towards developing a system that considers the needs and preferences of all.

Several areas potential areas for research were identified specifically from the men’s session. First the men identified long delays in accessing services. Delays are unfortunately not uncommon in the current climate of national healthcare in the UK post Covid-19 [22], and have undoubtably been exacerbated by NHS strike action and staffing problems, current at the time of writing. However, it might be worth exploring whether the delays described by the men in the WC are something that affects men universally in the Bristol region, or whether there are specific problems for men in the Somali community or other minoritised ethnic communities that need to be addressed, and if so, explore practical ways to do this. Second, some men described seeking healthcare abroad due to difficulties in accessing care in the UK. It could be valuable to investigate the prevalence of health tourism in this context due to the potential health and ethical implications of this.

### Outcomes taken forward because of the World Cafés

To further develop ideas for potential research projects stemming from these discussions, we are seeking funds to stage a ‘hack day’. This event will bring together community members, researchers, service providers and funders to discuss key messages and develop research projects.

The women from WC1 identified the need to improve health promotion within communities by providing education, training and support around mental health and wellbeing in ways that are accessible and understandable. Based on these discussions, we are co-developing an application for additional funding to co-produce learning resources about mental health and wellbeing through a series of workshops with these women.

Some of the topics identified in the World Café discussion such as miscommunication, cultural barriers to communication about prostate cancer, accessing GP services including testing and role of pharmacists in part informed the development of an NIHR grant application. Additionally, the men requested for an educational session from a clinician to raise awareness about prostate cancer, which we are in the process of organising.

### Strengths and weaknesses

By adapting and using this existing participatory approach to work with minoritised ethnic communities to explore research agenda setting, we have added valuable contributions to these practices. SD had built and sustained working relationships with various community leaders through previous research studies. This allowed us to work effectively with the community leaders in this public involvement work. We have shown how community leaders played a key role in co-delivering the workshops by recruiting participants through their existing relationships and facilitating the discussions in different languages. Both World Cafés were well attended with over 20 people attending each workshop. It was possible to identify important issues across the World Cafés and areas discussed, and as a result produce actionable outcomes that will be taken forward through further work. Other issues identified required follow-up with communities to explore appropriate routes to address them.

Working with community leaders and facilitators was central to the successful engagement with the communities in this project. However, this work also highlights areas for improvement. We noticed that discussions did not always align with the intended questions, for example, there could have been more focused discussion about family influences in seeking support in the men’s session; thus potentiallyindicating that the community were more reticent to talk about these aspects. Community facilitators could have benefitted from more training in facilitating the World Café sessions to ensure that more difficult areas are covered in the discussion. However, these sessions were also organised to be community led, enabling them to frame the discussion on their own terms, according to what is important to them. Although the World Cafés were well attended, this was not a research exercise, formal qualitative sampling techniques were not adopted, and theoretical saturation was not a goal for this work. Therefore, it cannot be assumed that other issues would not have been raised by people unable to attend. Finally, we did not conduct formal evaluations from the participants, community leaders or researchers involved to understand what worked well and what should be improved for future World Cafés . However, co-writing this paper with the community leaders involved has promoted a general reflection on the World Cafés and moving forward we would build this into future World Cafés and recommend this to others.

### Lessons learned

1. Community leaders play a key role in co-delivering workshops by recruiting participants and facilitating discussions in different languages.
2. The World Café approach can be effective in engaging underserved communities in discussions about research agendas and services.
3. It is possible to identify important research agendas across multiple workshops and areas discussed using the World Café approach.
4. Working with partner organisations is important to find the best way to take forwards issues raised by initiatives of this kind.
5. It is important to be realistic to members of the public participating in World Cafés about the potential limitations (e.g., barriers due to funding or spheres of influence) in developing initiatives (e.g., research projects or changes to service provisions) from the agendas they identified.

### Recommendations for future World Cafés with diverse communities

1. World Café focus and questions should be co-developed with communities and community leaders should play a key role in recruiting participants and co-delivering and facilitating the sessions.
2. Formal evaluations from participants are important to understand their perceptions of what did and did not work well, and how future World Cafés can be improved.
3. There is a need for follow-up with communities after initial World Cafés to explore appropriate routes to address the issues raised. In health and research agenda setting, this is likely to include developing networks between research organisations, and statutory and third-sector service providers.

## Conclusion

Our work has shown that the World Café method can be an effective way to engage with underserved communities. This paper has revealed some key agendas for future primary care research in the areas of mental health and prostate cancer through two World Cafés with women and men from minoritised ethnic communities. We have shown how this flexible and inexpensive participatory approach provides a safe space that encourages ideas generation and sharing of personal experiences, particularly when the sessions are co-developed and facilitated by community members. The discussions identified issues for further research and recommendations for service improvements that can be explored and developed through continued collaboration with communities. We would recommend others adopt similar approaches in their future practices to better understand the needs of communities in meaningful and actionable ways.

### Supplementary Information

**Additional file 1.** GRIPP 2 checklist.

## Data Availability

Not applicable as this was not a research study. We have shared the storyboards developed from the workshops.

## Abbreviations

NIHR: National Institute for Health and care Research
NIHR INCLUDE: National Institute for Health and care Research INnovations in CLinical trial design and delivery for the UnDEr-served
