# Correction to: World Cafés as a participatory approach to understanding research agendas in primary care with underserved communities: reflections, challenges and lessons learned

**DOI:** 10.1186/s40900-024-00592-0

**Published:** 2024-07-02

**Authors:** Carmel McGrath, Mari‑Rose Kennedy, Andy Gibson, Samira Musse, Zahra Kosar, Shoba Dawson

**Affiliations:** 1 NIHR Applied Research Collaboration West, University Hospitals Bristol and Weston NHS Foundation Trust, Bristol, UK; 2 NIHR Health Protection Research Unit in Behavioural Science and Evaluation, Population Health Sciences, Bristol Medical School, University of Bristol, Bristol, UK; 3 Faculty of Health and Applied Sciences, School of Health and Social Wellbeing, University of West England, Bristol, UK; 4 People in Health West of England, University of the West of England, Bristol, UK; 5 Barton Hill Activity Club, Bristol, UK; 6 Bristol Medical School, University of Bristol, Bristol, UK; 7 Somali Resource Centre, Bristol, UK; 8 Centre for Academic Primary Care, Bristol Medical School, University of Bristol, Bristol, UK

**Correction to:** **Res Involv Engagem 9, 101 (2023)**

**https://doi.org/10.1186/s40900-023-00509-3**

Following publication of the original article [1], the authors reported errors in the Funding section. The Wellcome funder grant number 204813/Z/16/Z was missing. The complete Funding section has been indicated hereafter.

The incorrect Funding section reads:
